# Pattern of cancer/testis antigen expression in lung cancer patients

**DOI:** 10.3892/ijmm.2012.896

**Published:** 2012-01-24

**Authors:** YEONG-DAE KIM, HAE-RIM PARK, MYUNG-HA SONG, DONG-HOON SHIN, CHANG-HUN LEE, MIN-KI LEE, SANG-YULL LEE

**Affiliations:** 1 Department of Thoracic Surgery, Pusan National University Hospital, Seo-gu, Busan 602-739; 2 Department of Biochemistry, School of Medicine, Pusan National University, Beomeo-ri, Mulgeum-eup, Yangsan 626-770; 3 Department of Pathology, School of Medicine, Pusan National University, Beomeo-ri, Mulgeum-eup, Yangsan 626-770; 4 Department of Internal Medicine, Pusan National University Hospital, Seo-gu, Busan 602-739, Republic of Korea

**Keywords:** lung cancer, cancer/testis antigen, cancer immunotherapy, MAGE-3, NY-SAR-35

## Abstract

Cancer/testis (CT) antigens represent promising targets for immunotherapy. We investigated the composite expression of 13 CT antigens by RT-PCR in 79 lung cancer tissues and by immunohistochemistry in 22 lung cancer tissues. In the 79 lung cancer tissues, MAGE-3 (42%) was expressed most frequently and followed by NY-SAR-35 (33%), NY-ESO-1 (30%), MAGE-1 (27%), CT-7 (20%), MAGE-4 (19%), LAGE-1 (16%), and MAGE-10 (14%). Twenty-one tissues did not express any of the CT antigens tested, 58 (73%) expressed at least one, 36 (46%) co-expressed two, 24 (30%) co-expressed three, 17 (22%) co-expressed four, 14 (18%) co-expressed five, 8 (10%) co-expressed six, 4 (6%) co-expressed seven and 2 tissues expressed 9 of the 13 examined CT antigens. Expression of CT antigens was significantly associated with age (P<0.001), smoking history (P=0.009), and gender (P=0.001) of patients, whereas no correlation was found between the expression of CT antigens and other clinical factors, such as pT status, pN status, tumor stage, and histology history. The present results show that CT antigens are potential candidates in lung cancer patients for polyvalent immunotherapy.

## Introduction

Lung cancer is the major cause of cancer-related mortality worldwide and is also one of the most frequent causes of death in Korea (1). The high mortality of lung cancer generally results from the difficulties in early diagnosis and the lack of effective therapeutic methods. Established therapeutic methods for lung cancer are surgical resection, chemotherapy, and radiotherapy. The results remain unsatisfactory, making it imperative that new diagnostic and therapeutic methods be developed. Immunotherapy is one of the alternative treatments for lung cancer, because various immunotherapies seem to improve the prognosis of patients with lung cancer (2).

An essential condition for the development of effective immunotherapeutic strategies is the existence and identification of tumor specific antigens that are either exclusively or preferentially expressed in malignant compared to normal tissues (3). Human tumor antigens are classified in several categories, including differentiation antigens (4), mutated gene products (5), overexpressed oncogenes (6), and cancer/testis antigens (CT) (7,8).

CT antigens are immunogenic proteins expressed in normal testis and in different types of tumors (3,9). CT antigens are promising candidates for cancer immunotherapy and the identification of novel CT antigens is a prerequisite for the development of cancer vaccines (10,11). CT antigens have previously been isolated by various methods. Examples of CT antigens are the T-cell defined MAGE (12), BAGE (13), and GAGE (14) antigens, as well as SSX-2 (15), NY-ESO-1 (16), SCP-1 (17), CT-7 (8), NY-SAR-35 (18), and NY-TLU-57 (19), all which have been defined using SEREX (the serological identification of antigens by recombinant expression cloning) (20). To date, more than 100 CT antigens have been identified and their expression studied in numerous cancer types (11). Only 19 protein products of CT antigen families have been demonstrated to be able to elicit an immune response in humans (3,11). Though little is known about the biological function of CT antigens, knowledge about their presence in lung cancer tissues can have important implications for the understanding of the biology of both lung cancer and cancer immunity. Previous analysis of the CT antigens revealed that some of them are also expressed in lung cancer (21,22). However, little is known about analysis of a larger panel of CT antigens especially in lung cancer tissues.

The present study analyzes the frequency of expression of 13 CT antigens in 79 lung cancer tissues. The correlation between CT antigen expression patterns and pathological characteristics of lung cancer tissues was also studied.

## Materials and methods

### Lung cancer tissues

Human tumor tissues were obtained from lung cancer operations performed at the Pusan National University Hospital, Busan, Korea. Analyses were performed on tumor tissue samples of 79 patients with lung cancer confirmed pathologically. The tumor samples consisted of 33 cases of adenocarcinoma, 24 of squamous cell carcinoma, 6 of neuroendocrine carcinoma, 4 of pleomorphic carcinoma, and 12 of others (3 adenosquamous carcinoma, 2 unclassified non-small cell carcinoma, 2 small cell carcinoma, 1 small round cell sarcoma, and 4 metastatic cancers). Among the 79 lung cancer patients from whom tissue samples were obtained, 68 had available records, but 11 did not. Data for the 68 tissues including gender, age, classification of TNM status, and stages were obtained from the clinical and pathological records. Tumor stage and progression were classified according to the International Staging System (23).

### Total-RNA extraction from lung cancer tissues

Total cellular RNA was extracted from frozen tissue specimens of 79 lung cancer samples, using TRI Reagent (Molecular Research Center, Inc.). RNA extraction using TRIzol (Invitrogen Life Technologies, Carlsbad, CA), a common protocol, was used. Tumor tissues removed at surgery were snap-frozen and stored at −70°C. Total-RNA was isolated from ~100 mg of each tissue sample using 1 ml TRI Reagent (Molecular Research Center, Inc.), extracted with chloroform, precipitated with isopropyl alcohol, washed with ethanol and re-dissolved in RNase-free water. The amount of isolated RNA was measured by a spectrophotometer (Ultrospec 2000, Pharmacia Biotech) at 260 nm.

### Reverse transcription (RT)-PCR

The cDNA preparations used as templates in the RT-PCR reactions were prepared by using 500 ng of total-RNA in conjunction with the SuperScript First Strand Synthesis kit (Invitrogen Life Technologies).

The 13 primer sets for the 13 CT antigens and the lengths of each PCR product are shown in Table I. Each 20 μl PCR mixture consisted of 2 μl cDNA, 0.4 μl of 10 mM dNTP-mix (Solgent), 2 μl of 10X Taq buffer (Solgent), gene specific forward and reverse primers, and 0.2 μl of TaqDNA polymerase (Solgent). For PCR, DNA polymerase activation was performed for 5 min at 94°C, and then amplification was performed in a 96-well Gene Amp PCR System 9700 for 35 cycles as follows: 1 min at 94°C; 1 min at the respective annealing temperature as indicated in Table I; 1 min at 72°C and was concluded with a final extension step of 10 min at 72°C. A 20 μl aliquot of each reaction was size-fractionated on a 1.5% agarose gel, visualized by ethidium bromide staining and assessed for products of the expected size.

### Immunohistochemistry

The M3H67 monclonal antibody (to MAGE-3) was obtained from the Ludwig Institute for Cancer Research, New York Branch at the Memorial-Kettering Cancer. Immunohistochemical staining was performed according to the previously report (24). Briefly, paraffin sections were applied to slides for immunohistochemistry and heated for 20 min at 60°C. Slides were deparaffinized and rehydrated in a series of graded alcohols. Antigen retrieval was performed by placing the slides in TE buffer (pH 9.0) and heating for 40 min in a steamer and then allowed to cool. M3H67 of 1.0 μg/ml antibody was incubated for 1 h in a room temperature. Sections were washed with PBS for 20 min and then slides were incubated with a secondary antibody for 1 h at 37°C, washed and incubated with the avidin-biotin complex system for 1 h at 37°C. Sections were sequentially washed and then slides were counterstained with hematoxylin. The extent of staining was estimated and graded as follows: focal staining of single cells or small clusters (>50% total) was considered positive, whereas focal staining of single cells or small clusters (<50% total) was considered negative.

### Statistical analysis

Statistical analysis was performed with the SPSS program (version 11.5; SPSS Inc., Chicago, IL). Pearson χ^2^ test was used to compare the correlation between disease stage, grade, and CT antigen expression. Statistical significance was accepted at P<0.05.

## Results

### Expression of CT antigens in lung cancer tissues

**Figure 1:**
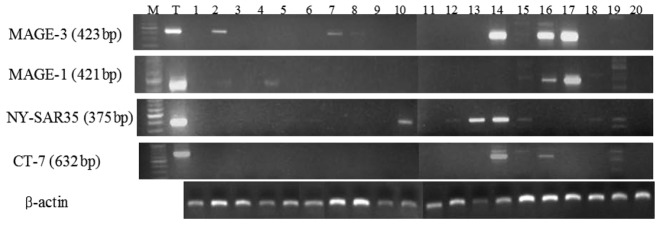
Representative RT-PCR results of the analysis of MAGE-3, MAGE-1, NY-SAR-35, and CT-7 expression in lung cancer tissues. β-actin was used as internal quality control (approximate length, 100 bp). T, normal testis used as a positive control; M, marker.

**Figure 2:**
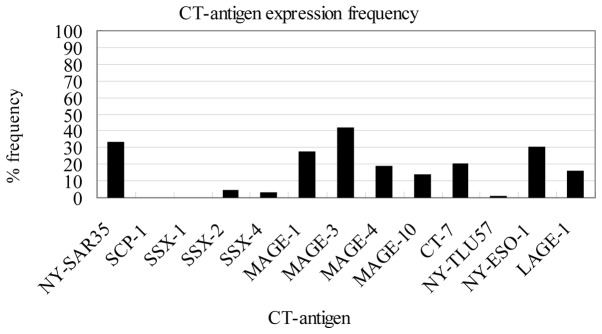
Frequency of CT antigen expression in lung cancer tissues. The proportion of RT-PCR positive cases is shown as a percentage of 79 evaluable lung cancer tissues. The most frequently expressed CT antigen was MAGE-3 (33/79, 42%), followed by NY-SAR-35 (26/79, 33%), NY-ESO-1 (24/79, 30%), and MAGE-1 (21/79, 27%).

Expression of the 13 CT antigens (NY-SAR-35, SCP-1, SSX-1, SSX-2, SSX-4, MAGE-1, MAGE-3, MAGE-4, MAGE-10, CT-7, NY-TLU-57, NY-ESO-1 and LAGE-1) was assessed by RT-PCR in 79 lung cancer tissue samples. Representative RT-PCR results from lung cancer tissues are shown in Fig. 1. The most frequently expressed CT antigen was MAGE-3 (33/79, 42%), followed by NY-SAR-35 (26/79, 33%), NY-ESO-1 (24/79, 30%), MAGE-1 (21/79, 27%), CT-7 (16/79, 20%), MAGE-4 (15/79, 19%), LAGE-1 (13/79, 16%), MAGE-10 (11/79, 14%), SSX-2 (3/79, 4%), SSX-4 (2/79, 3%), NY-TLU-57 (1/79, 1%), SCP-1 (0/79, 0%) and SSX-1 (0/79, 0%) (Fig. 2). In previous studies, the MAGE-3 antigen was expressed in between 30–50% of the lung cancer tissues examined (25,26). In the present study, MAGE-3 was detected in 42% of all lung cancer tissue samples. Among the 58 CT antigen-positive lung cancer tissue samples, MAGE-3 and NY-SAR-35 were respectively expressed in 57 and 45% of the samples. These findings indicate that MAGE-3 and NY-SAR-35 are attractive targets for antigen-specific immunotherapy in Korean lung cancer patients.

### Co-expression of multiple CT antigen mRNA in lung cancer tissues

**Figure 3:**
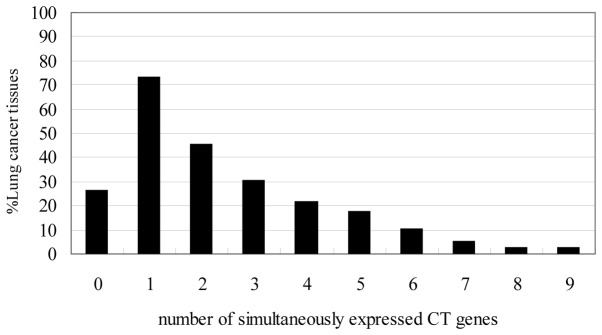
Simultaneous expression of multiple CT antigens in lung cancer tissues. The proportion of lung cancer tissues expressing at least one and up to 13 CT antigens is shown as a percentage of 79 lung cancer tissues. Of the 79 lung cancer tissues investigated, only 21 (26%) did not express any of the CT antigens examined, 58 lung cancer tissues (73%) expressed at least one and 36 cases (45%) expressed more than two CT antigens.

The percentages of co-expressed CT antigens in lung cancer tissues are shown in Fig. 3. Fifty-eight of 79 lung cancer tissues (73%) were found to express at least one of the CT antigens, whereas 21 (26%) did not express CT antigens. Thirty-six cases (45%) expressed more than two CT antigens and three or more CT antigens were expressed in 24 cases (30%). Seventeen specimens expressed ≥4 (21%), 14 specimens expressed ≥5 (18%), 8 cases ≥6 (10%), 4 cases ≥7 (5%), 2 cases ≥8 (3%) and two tissues co-expressed 9 antigens. From these data, it becomes evident that 73% of our patients with lung cancer tissues would be eligible for antigen specific immunotherapeutic approaches with at least one CT antigen.

### Relationship between cancer/testis antigen expression and tumor characteristics

The characteristics of the patients are summarized in Table I. The following data of the patients were entered in a prospective database: there were 49 men and 19 women, with ages ranging from 37 to 80 years (>60 vs. ≤ 60 years of ages); 22 patients were non-smokers, and 46 patients were smokers; there were 26 patients with pT1, 35 with pT2 and 7 with pT3; there were 54 patients with pN0 or pN1 and 14 patients with pN2 or pN3. In addition, there were 50 patients with clinical stage I or II and 18 with clinical stage III or IV at the time of diagnosis. We then investigated the possible correlation between CT antigen expression and these clinical variables (Table II). The data included patients whose tumors expressed at least one CT antigen. CT antigen expression was found to be associated with male gender (P=0.001), age (P<0.001), and smoking history (P=0.009). On the other hand, no correlation was detected between CT antigen expression and other clinical factors, such as pT status, pN status, tumor stages, and histology history. Between the adenocarcinoma and squamous cell carcinoma samples, expression of CT antigens in adenocarcinoma samples had a tendency to be more frequent than in squamous cell carcinoma. In addition, expression of individual MAGE-3 (P=0.003) and NY-SAR-35 (P=0.036) were found to be associated with squamous cell carcinoma and adenocarcinoma, respectively (Table III).

### MAGE-3 protein expression

**Figure 4:**
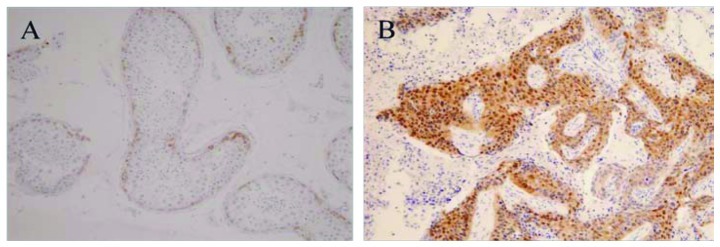
Immunohistochemical staining of MAGE-3 antigen with the mAb M3H67. (A) The expression of MAGE-3 protein is identified in the normal testis and (B) in moderately differentiated lung squamous cell carcinoma. In both cases antibody binding is indicated by brown staining. (magnification, ×200).

Among the 79 lung cancer tissue specimens, 22 lung carcinoma samples from which sufficient material was available were investigated for the expression of the frequently expressed MAGE-3 protein by immunohistochemistry. Representative immunohistochemistry results of lung cancer tissues with the mAb MAGE-3 are shown in Fig. 4. Expression of MAGE-3 protein was found in 12 of 22 tumor samples (55%) while MAGE-3 mRNA expression was detected in 10/22 tumor samples (45%). Expression of MAGE-3 antigen was observed in 7 of 10 MAGE-3 RT-PCR-positive tumor samples. We observed a correlation between the expression of MAGE-3 protein and the histological type of lung cancer tissues. The MAGE-3 protein was expressed in 8 of 10 squamous cell carcinomas as compared with 3 of 10 adenocarcinomas.

## Discussion

CT antigens are immunogenic proteins expressed in normal testis and in different types of tumors. Because of their tissue-restricted expression, CT antigens are ideal candidates for antigen-specific cancer immunotherapy. In general, CT antigens are expressed in 20–40% of specimens from a given tumor type (3). The present study was undertaken to evaluate the expression of CT antigens (NY-SAR-35, SCP-1, SSX-1, SSX-2, SSX-4, MAGE-1, MAGE-3, MAGE-4, MAGE-10, CT-7, NY-TLU57, NY-ESO-1, and LAGE-1) in lung cancer tissues. Moreover, the prognostic role of CT antigen expression and their correlation with a number of clinical pathological parameters were examined. A number of studies have investigated the mRNA expression patterns of individual CT antigens in lung cancer (19,25,27,28). In this study, the SEREX-defined (33% NY-SAR-35 and 30% NY-ESO-1) and the CTL-defined (42% MAGE-3 and 27% MAGE-1) antigens were the most frequently expressed in lung cancer tissues. As MAGE-3, NY-SAR-35, NY-ESO-1 and MAGE-1 were expressed with a high percentage and specificity in lung cancer tissues, their products might be ideal for antigen targets for lung cancer immunotherapy (Fig. 2).

In addition, 58 of 79 lung cancer tissue specimens (73%) were found to express at least one of the CT antigens. According to our results, 73% of our lung cancer patients would be eligible for specific immunotherapeutic approaches with at least one CT antigen. A possibility for the high observation frequency of CT antigen expression in lung cancer tissues could be due to the characteristics of the patients. These results indicate that expression of CT antigens was significantly associated with the age and gender of the patients, whereas no significant correlation was detected between CT antigen expression and other clinical factors such as pT status, pN status, and tumor stages (Table II). In this study, the differences in the expression of CT antigens in 79 lung cancer tissues cannot be ascribed to the characteristics of the patients, but rather reveal intrinsic differences in lung cancer tissues.

The highly homologous NY-ESO-1 and LAGE-1 (94%, nucleotide identity and 88% amino acid identity) are highly immunogenic, and a CTL response to an immunodominant, HLA-A2-restricted NY-ESO-1/LAGE-1 epitope can commonly be detected in cancer patients (29,30). We compared the expression of NY-ESO-1 and LAGE-1 in tissues from lung cancer patients by RT-PCR (Figs. 2 and 3). NY-ESO-1 gene expression was detected in 24/79 (30%) more frequently than in two previous reports, in which NY-ESO-1 was detected in 2/12 (17%) (16) and 3/15 (20%) (31) lung cancer tissues of Caucasian origin. LAGE-1 gene expression was detected in 13/79 (16%) specimens, less frequently than in a previous report (31) in which it was detected in 5/15 (33%) cases. Of the 79 lung cancer tissues, 24/79 (30%) were NY-ESO-1-positive by RT-PCR and 13/79 (16%) were LAGE-1 positive by RT-PCR. The expression of either NY-ESO-1 or LAGE-1 antigens was observed in 29 of 79 (37%) of lung cancer tissue specimens. These results suggested that NY-ESO-1 and LAGE-1 represent targets for immunotherapy in a significant proportion of patients with lung cancer.

Of particular interest was NY-SAR-35, which represents a CT antigen as it appears to be a rare example of a cell surface antigen (19), which was highly expressed in lung cancer tissues. Expression of NY-SAR-35 was found in 33% of all lung cancer tissues. NY-SAR-35 was detected in 45% of 58 CT antigen-positive lung cancer tissues. Recently, we found that treatment with 5-aza-CdR can induce the expression of NY-SAR-35, and that transcriptional silencing of NY-SAR-35 is caused by hypermethylation of its promoter (32). These findings indicated that NY-SAR-35 is an attractive target for antigen-specific immunotherapy in lung cancer and that treatment with demethylating agents, in combination with immunotherapy, could be a useful therapeutic strategy for modulating the antigen expression.

To confirm the existence of MAGE-3 protein expression, 22 lung carcinoma samples from which sufficient material was available were immunohistochemically stained by a specific MAGE-3 monoclonal antibody. Similarly to our previous study (24), we found a correlation with frequent expression of MAGE-3 protein and the histological type of squamous cell carcinomas. The MAGE-3 protein was expressed in 8 of 10 squamous cell carcinomas as compared with 3 of 10 adenocarcinomas. Expression of MAGE-3 protein was demonstrated in 7 of 10 MAGE-3 RT-PCR-positive tumor samples. These results indicate the discordance between RT-PCR and immunohistochemistry positivity. As a note of caution, however, one should keep in mind that MAGE-3 at the protein level may not be detected in all cases expressing the respective mRNA. Whether this is due to a lower sensitivity of the detection method used (immunohistochemistry using CT antigen-specific antibodies) or whether the mRNA is not translated into protein cannot be determined at this point (27,33).

From the data of our study, a high proportion (58/79, 73%) of lung cancer tissues was positive for at least one of these 13 CT antigens as targets will greatly increase the number of candidates for CT antigen-based lung cancer immunotherapy. However, our results showed no expression of CT antigens in 27% (22/79) of lung cancer tissues. For these patients, it is necessary to screen other CT antigens or tumor-specific antigens serving as immune targets for lung cancer tissue immunotherapy. In conclusion, this study demonstrated that lung cancer tissues frequently express CT antigens and a high percent express more than one CT antigen, suggesting that CT antigens are potential candidates for polyvalent immunotherapy.

## Figures and Tables

**Table I:** Primers used for RT-PCR.

Genes	Primer sequences	Annealing temperature (°C)	References
NY-SAR-35	F: 5′-CTTGGTGCGATCAGCCTTAT-3′		
	R: 5′-TTGATGCATGAAAACAGAACTC-3′	55	(18)
SCP-1	F: 5′-GTACAGCAGAAAGCAAGCAACTGAATG-3′		
	R: 5′-GAAGGAACTGCTTTAGAATCCAATTTCC-3′	60	(18)
SSX-1	F: 5′-CTAAAGCATCAGAGAAGAGAAGC-3′		
	R: 5′-AGATCTCTTATTAATCTTCTCAGAAA-3′	60	(18)
SSX-2	F: 5′-GTGCTCAAATACCAGAGAAGATC-3′		
	R: 5′-TTTTGGGTCCAGATCTCTCGTG-3′	65	(18)
SSX-4	F: 5′-AAA TCGTCTATGGTATATGAAGCT-3′		
	R: 5′-GGGTCGCTGATCTCTTCATAAAC-3′	60	(18)
MAGE-1	F: 5′-GCTGGAACCCTCACTGGGTTGCC-3′		
	R: 5′-CGGCCGAAGGAACCTGACCCAG-3′	62	(18)
MAGE-3	F: 5′-GAAGCCGGCCCAGGCTCG-3′		
	R: 5′-GGAGTCCTCATAGGATTGGCT-3′	62	(18)
MAGE-4	F: 5′-GAGCAGACAGGCCAACCG-3′		
	R: 5′-AAGGACTCTGCGTCAGGC-3′	65	(18)
MAGE-10	F: 5′-GGAACCCCTCTTTTCTACAGAC-3′		
	R: 5′-TCCTCTGGGGTGCTTGGTATTA-3′	60	(18)
CT-7	F: 5′-GACGAGGATCGTCTCAGGTCAGC-3′		
	R: 5′-ACATCCTCACCCTCAGGAGGG-3′	60	(18)
NY-TLU-57	F: 5′-TCATATGCCTAGCTCTGTCAAAAG-3′		
	R: 5′-TCCCGGGTCTGGCATCAATAAAAT-3′	60	(19)
NY-ESO-1	F: 5′-CCCCACCGCTTCCCGTG-3′		
	R: 5′-CTGGCCACTCGTGCTGGGA-3′	60	(19)
LAGE-1	F: 5′-CTGCGCAGGATGGAAGGTGCCCC-3′		
	R: 5′-GCGCCTCTGCCCTGAGGGAGC-3′	62	(19)

Primers were referenced from http://www.cancerimmunity.org/CTdatabase.

**Table II:** Summary of patient characteristics and expression of CT antigens.

Characteristic	Number of patients	Number of patients expressing CT gene mRNAs	P-value
Age			
>60	29	11	<0.001
≤60	39	35	
Gender			
Male	49	42	0.001
Female	19	8	
Smoking history			
No	22	11	0.009
Yes	46	38	
pT status			
pT1	26	19	0.678
pT2	35	25	
pT3 and 4	7	6	
pN status			
pN0 and 1	54	39	0.295
pN 2 and 3	14	11	
Pathological stage			
I and II	50	36	0.635
I and IV	18	14	
Histology			
Adenocarcinoma	33	25	0.55
Squamous cell carcinoma	24	16	
Neuroendocrine carcinoma	6	6	
Carcinoma with pleomorphic	4	3	
Others	12	8	

**Table III:** The expression of CT antigens according to the histological classification of lung cancer tissues.

		Number of patients expressing a CT gene												
		
Histological type	No. of patients	NY-SAR35	SCP-1	SSX-1	SSX-2	SSX-4	MAGE-1	MAGE-3	MAGE-4	MAGE-10	CT-7	NY-TLU57	NY-ESO-1	LAGE-1
Adenocarcinoma	33	10	0	0	1	1	4	8	3	3	6	0	8	4
Squamous cell carcinoma	24	4	0	0	1	0	7	13	9	4	4	0	7	3
Neuroendocrine carcinoma	6	4	0	0	1	0	4	5	1	2	3	1	3	3
Sarcomatoid carcinoma	4	3	0	0	0	1	3	4	1	2	2	0	3	0
Others	12	4	0	0	0	0	3	2	0	0	1	0	2	3
P-value^a^		0.036					0.010	0.003	0.049	0.094	0.217		0.236	0.072

a Statistical analysis was performed for the CT antigen groups in which >5 specimens were positive.
